# Targeting macrophage phenotypes to prevent diseases caused by *Leishmania* and *Trypanosoma cruzi* infections

**DOI:** 10.3389/fimmu.2025.1595954

**Published:** 2025-08-07

**Authors:** Natália S. Vellozo, Thayane C. Matos-Silva, Marcela F. Lopes

**Affiliations:** Laboratório de Biologia Imunitária George DosReis, Instituto de Biofísica Carlos Chagas Filho, Universidade Federal do Rio de Janeiro, Rio de Janeiro, RJ, Brazil

**Keywords:** ATRA, Axl, Chagas disease, efferocytosis, Leishmaniasis, M1 and M2 macrophages, RANKL, Th1 and Th2 cytokines

## Abstract

Macrophage plasticity is remarkable, and recent studies have opened new prophylactic and therapeutic avenues for immunomodulation of macrophage phenotypes in inflammatory and infectious diseases. During infections caused by the pathogenic protozoans *Leishmania* spp. and *Trypanosoma cruzi*, susceptibility to disseminated or chronic infections and/or the development of inflammatory diseases depend on the balance between protective immunity mediated by macrophages and anti-inflammatory responses. Here, we will discuss strategies that exploit macrophage plasticity towards the extreme proinflammatory M1 or pro-infection M2 phenotypes to prevent the establishment of disseminated and chronic infection or to temper parasite-driven inflammatory responses. Immunomodulation of macrophage phenotypes has been tested in experimental models of protozoan infections through pharmacological approaches, synergy between pro-M1 cytokines, and targeting of pro-M2 macrophage functions, such as efferocytosis. We will address the cellular and molecular mechanisms underlying strategies designed to redirect macrophage activation towards M1 and M2 phenotypes, as well as the challenges and open questions.

## 1 Introduction

The pathogenic protozoans *Trypanosoma cruzi* (1) and *Leishmania* spp. (2) cause, respectively, Chagas disease and the Leishmaniasis spectrum, which challenge Public Health systems worldwide and afflict impoverished populations (3, 4). Vector-borne *Leishmania* parasites establish localized infection and lesion in the skin or reach mucosa and target organs, such as liver and spleen, or yet disseminate systemically, causing different pathologies referred to as Leishmaniasis (2). Although other host cells have been described (5), macrophages are the preferential host cells for *Leishmania* spp. and their ability to contain phagocytosed parasites or otherwise to fuel intracellular infection depends both on the host immune system and pathogen molecules that induce or subvert protective macrophage-mediated responses (6–8).

*T. cruzi* parasites spread from the initial focus of vector-transmitted infection through the blood to reach multiple tissues, where they invade cell cytoplasm, replicate and induce rupture of fibroblasts, myocytes, macrophages, and other cells (9, 10). In addition to host *T. cruzi* parasites, macrophages play multiple roles in the immune response, by inducing inflammation and by harvesting cell debris, apoptotic cells and parasites released by other cells (11–13). Therefore, how macrophages deal with infection determines the extension of parasite spread to other cells/tissues, leading to the development of chronic infection and Chagas disease after multiple rounds of parasite-driven inflammation, especially in the heart (10–12, 14).

**Figure 1:**
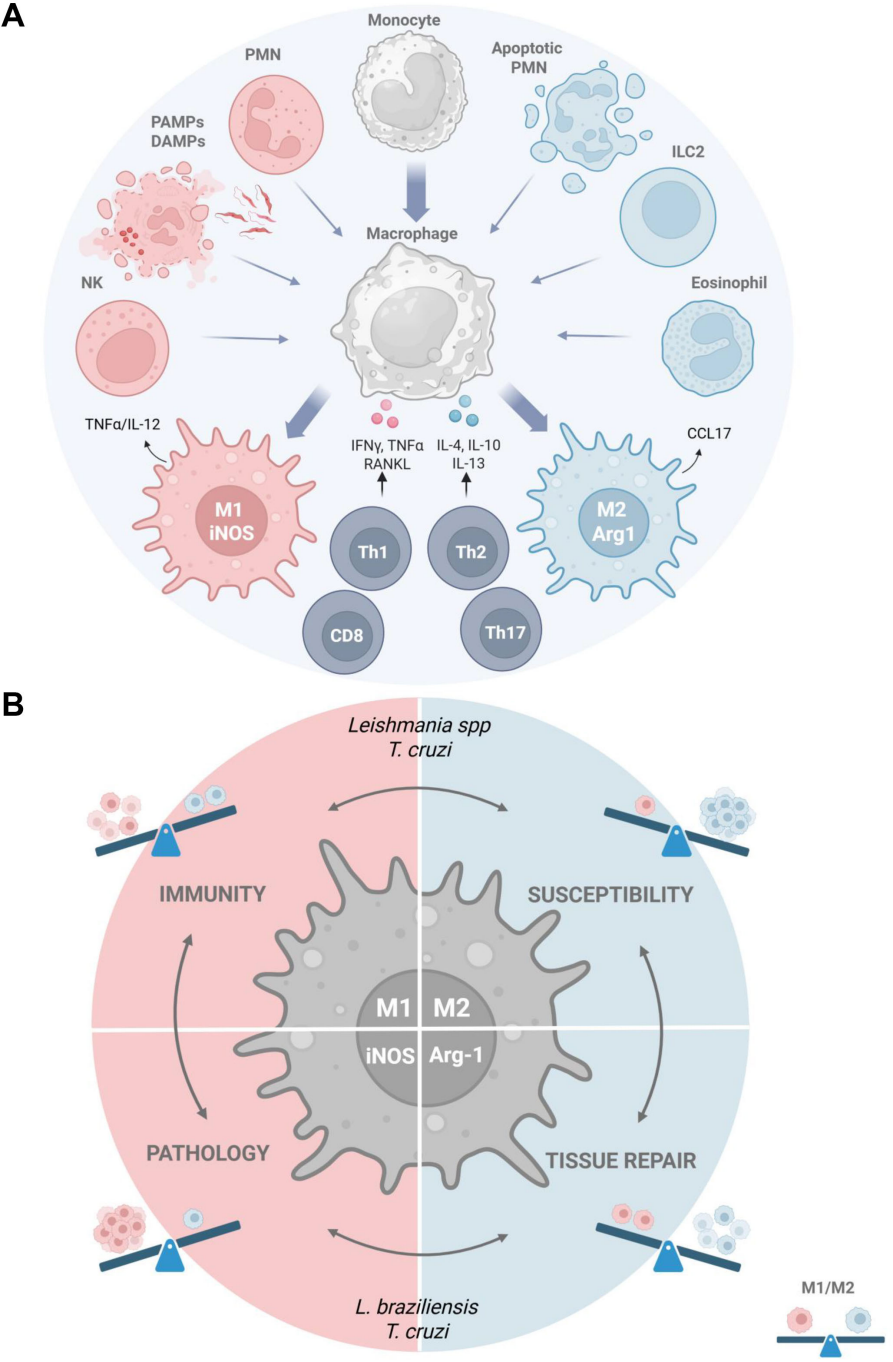
Targeting macrophage plasticity in parasite infection. **(A)** Tissue environment shapes macrophage differentiation towards M1 and M2 phenotypes. Upon parasite infection, monocytes generate inflammatory macrophages which depending on environmental stimuli differentiate into M1 that express iNOS, NO, and kill parasites or into pro-tissue repair M2 that express Arg1 and host parasites. Parasite PAMPs and DAMPs from disrupted infected cells, PMNs, and NK cells induce M1, whereas apoptotic cells, eosinophils, and ILC2 lead to alternative activation of macrophages toward M2 responses. In addition to innate immunity, T cells modulate M1 and M2 phenotypes and establish T cell-macrophage crosstalk that involves both cell surface ligands and secreted cytokines, such as IFN-γ and IL-4 to promote adaptive immunity. **(B)** During *Leishmania* spp. and *T. cruzi* infection, classically activated (M1) macrophages control infection but also induce exacerbated inflammatory responses that lead to pathology, whereas alternatively activated (M2) macrophages promote intracellular infection and/or tissue repair. The possible outcomes of modulation targeting macrophage plasticity include but are not restricted to protective immunity, exacerbated infection, pathology, and tissue remodeling depending on the direction and intensity of environmental stimuli. Created with BioRender.com. Arg1, arginase 1; CCL, C-C motif chemokine ligand; DAMP, damage-associated molecular pattern; IL, interleukin; ILC2, type 2 innate lymphoid cells; iNOS, induced NO synthase; IFN-γ, interferon-γ; M1, Macrophage 1; M2, Macrophage 2; NK; Natural Killer cells; PAMP, pathogen-associated molecular pattern; PMN, polymorphonuclear cells; RANKL, the receptor activator of Nuclear Factor-κB ligand; Th, T helper cells; TNF-α, tumor necrosis factor-α.

Macrophages are functionally plastic in response to environmental stimuli, such as parasite PAMPs (pathogen-associated molecular patterns), cytokines, tissue-derived DAMPs (damage-associated molecular patterns), and apoptotic cells, by ranging from pro-inflammatory M1 macrophages, which fight infection, to pro-tissue repair M2 macrophages that eventually promote parasite replication (15–19) (**Figure 1A**). Here we will discuss how host-directed therapies can modulate the balance between M1 and M2 macrophages (20) to prevent the pathogenic outcomes of protozoan infections caused by *Leishmania* spp. and *T. cruzi* (**Figure 1B**).

## 2 The control of macrophage plasticity in protozoan infections

Experimental Leishmaniasis is the prototype model where Th1 and Th2 responses mediated by IFN-γ and IL-4 correlate with genetic resistance and susceptibility to *Leishmania major* in different mouse strains, i.e. C57BL/6 (B6) and BALB/c, respectively (21, 22). Macrophages exposed to Th1 cytokines and PAMPs were described as classically activated (M1) macrophages able to produce NO and fight infection, whereas Th2 cytokines, such as IL-4, IL-10 and IL-13 (23) induce alternatively activated (M2) macrophages, which express Arginase 1 (Arg1) and metabolize L-arginine towards the polyamine pathway (15, 16, 24–26). In addition to experimental models that develop Th1 or Th2 responses (22) and *in vitro* settings that generate polarized M1 or M2 macrophages (16), T cell and macrophage responses to protozoan infections show multiple/intermediate phenotypes between the extreme poles, especially within the M2 spectrum (18, 24). Here, we will not use the M1 and M2 terms to designate the strict phenotypes (16), but as a ‘compass’ to guide discussion on the immunomodulation towards M1 and M2 responses.

Both adaptive immunity (22, 23) and innate immunity (7, 27, 28) influence macrophage phenotype during infection and increase resistance or contribute to the development of disease. *Leishmania braziliensis*- but not *L. major*-recruited monocytes develop early M1 responses in the peritoneum of BALB/c mice (29). However*, L. braziliensis* induced a more efficient M1 response in B6 than in BALB/c mice, characterized by increased expression of the M1 hallmarks IL-12, induced NO synthase (iNOS), and NO production (29). These and other (30–32) experiments indicate that both parasite species and genetic backgrounds are relevant for macrophage responses during innate immunity. Exacerbated M1 responses may correlate with BALB/c resistance to *L. braziliensis* versus *L. major* infection (33) and the development of inflammatory disease underlying human mucocutaneous Leishmaniasis (34–36). Conversely, a series of studies support the deleterious role of M2-like monocytes and macrophages, which are better host cells for *Leishmania* parasites (37–43). Dermal-resident macrophages express M2 hallmarks and host *Leishmania* infection even in a mixed IFN-γ/IL-4 environment (27, 40, 44, 45). IL-4 from eosinophils contributes to maintenance of M2-like macrophages in a *Leishmania* infection model (44). Contrary to the Th1/Th2 paradigm, however, IFN-γ can increase the recruitment of M2-like monocytes that express Arg1 activity and promote parasite infection (41). Overall, M1 and M2 macrophages play a key role in resistance and susceptibility to *Leishmania* infection either in coordination with Th1 and Th2 responses or in a complete independent or unexpected fashion (15, 22, 23, 28, 41).

During *T. cruzi* infection, both innate and adaptive immunity induce M1 microbicidal macrophages that help to control infection, as evidenced by increased parasitemia and mortality in macrophage-depleted mice (46) or in mice bearing IFN-γ-signaling deficient macrophages (47). Natural Killer cells, CD4 and CD8 T cells produce IFN-γ (13, 48) and help macrophage activation into NO/iNOS-expressing M1 macrophages which are able to kill *T. cruzi* parasites and reduce further parasite-driven pathogenesis (11). The absence of M1 features, such as IL-12, leads to increased differentiation of M2 macrophages that propagate parasite infection and contribute to the development of Chagas disease (49). We previously discussed the role of M1 and M2 responses (11, 28) and their relevance in resistance and susceptibility to parasite-driven neglected diseases, where immunomodulation might add new therapeutic avenues to the insufficient treatment/vaccine portfolio (8, 50–52). Other discussions are available for comprehensive review (7, 11, 18, 28) and correlation with human diseases (53, 54). Here, we will focus on the experimental models that used host-directed therapies, such as mimicking T-cell macrophage cytokine crosstalk and synergy with Th1 and Th2 cytokines to induce M1 and M2 phenotypes, pharmacological interventions targeting induction/function of M1 and M2 macrophages, and identification of new pro-M2 molecular targets.

## 3 RANKL helps to induce M1 macrophages by mimicking T-cell macrophage crosstalk

In addition to the Th1/Th2 axis, the crosstalk between macrophages and T cells might involve other cytokines and ligands (23), such as IL-17, as discussed elsewhere (55) and the Receptor Activator of Nuclear Factor-κB Ligand (RANKL). RANKL, also known for its pro-osteoclastic properties, is a potential vaccine adjuvant that activate dendritic cells and macrophages to improve T cell proliferation and Th1 responses (56, 57). Moreover, RANKL may synergize with Th1 and Th2 environments to induce M1 and M2 macrophages, respectively (58, 59). In the context of Th1 macrophage crosstalk, T cells from *L. major*-infected B6 mice induce M1 responses in parasite-recruited monocytes in an antigen, RANKL and IFN-γ dependent manner (60). Whereas IFN-γ alone promotes TNF-α production in parasite-stimulated cocultures, neutralization of either IFN-γ or RANKL precludes IL-12 responses (60). To dissect how RANKL might promote M1 responses, we showed that thioglycolate-induced inflammatory macrophages express the receptor RANK and shift from M2 to M1 phenotype upon treatment with suboptimal IFN-γ concentration in the presence of RANKL (60). Low IFN-γ dose/RANKL-induced M1 macrophages express IL-12p35, iNOS, but reduced M2 features, such as Arg1, MR (mannose receptor) MGL (galactose-type lectin), and CCL17 (60). IFN-γ and RANKL synergism induces M1 responses, such as NO production and IL-12 secretion, through the NF-κB signaling pathway (60). Furthermore, low IFN-γ dose and RANKL promoted *L. major* control by macrophages in a ROS and NO-dependent fashion (60).

Multiple T-cell help mechanisms are probably redundant and CD40L deficient mice remain resistant to low numbers of *L. major* parasites in the B6 genetic background (61). However, blockade of RANKL in *L. major*-infected CD40L deficient mice prevented lesion healing, providing evidence that RANKL is necessary for T-cell DC crosstalk, IL-12 production, and Th1 responses (62). Accordingly, RANKL has been tested as an adjuvant for treating Ag-loaded DCs to improve Th1 responses (56) and as a vaccine-associated RANKL gene to induce anti-*T. cruzi* CD8 T cells (63). Interestingly, only a less virulent *T. cruzi* strain induced RANKL signaling pathway (64), which might contribute to M1 responses and control of infection, whereas more virulent strains subvert protective responses. Therefore, RANKL delivered locally is a safer prophylactic/therapeutic strategy that might help to improve immunity to protozoan parasites without disrupting bone homeostasis.

Other potential adjuvants, such as the cytokines APRIL (a proliferation-inducing ligand) and BAFF (B-cell activating factor), produced by DCs and monocytes, can improve M1 responses through interactions with their receptor TACI (transmembrane activator and a CAML interactor) (65). Although there are still open questions, such as how intracellular TACI receptor is mobilized to interact with the ligands, APRIL and BAFF signal through TACI receptor in macrophages to induce M1 responses and potentiate the control of *Leishmania* infection (65). Therefore, APRIL and BAFF are potential therapies and vaccine adjuvants to improve immunity in parasite infections.

## 4 Targeting M1 to M2 shift in protozoan infections

Exacerbated Th1/M1 responses underly or at least might contribute to severe outcomes in inflammatory diseases caused by protozoan parasites (36). In this sense, diversion from the proinflammatory M1 towards M2 phenotype is a potential therapeutic strategy. By dissecting the role of monocytes in *Leishmania* infection, we found that treatment with all-*trans*-retinoic acid (ATRA) promotes macrophage maturation at the cost of effective M1 responses (66). Whereas ATRA injection helps T cell proliferation by reducing immature myeloid cells-mediated suppression, early treatment with ATRA also reduced NO production and increased parasite load in lymph nodes of *L. major*-infected B6 mice (66). The effects of ATRA injection on monocyte phenotype can be adaptive immunity independent as showed in B6 or BALB/c mice treated with ATRA 24 h after i.p. *L. major* infection and analysed for immune responses 24 h later (29). Treatment with ATRA reduced M1 features, such as iNOS expression, IL-12 and TNF-α secretion, and increased parasite load within peritoneal macrophages (29). For comparing the direct effects of ATRA in BALB/c and B6 bone-marrow derived macrophages (BMDMs), we used an LPS (lipopolysaccharide)/cytokine setting that mimics a mixed Th1/Th2/infection environment (29). Treatment with ATRA reduced LPS-induced M1 hallmarks, such as secretion of TNF-α and CXCL9, and increased the M2 chemokines CCL17 and CXCL13. Moreover, ATRA downmodulated iNOS expression and NO production by LPS-stimulated macrophages (29). Whereas ATRA treatment might be deleterious by increasing susceptibility to *L. major* infection, it is reasonable to envision that ATRA could attenuate exacerbated M1 pathogenic responses (28, 36) and prevent parasite-driven inflammation and pathology upon pro-M1 *L. braziliensis* infection. More proof-of-principle studies are necessary for guiding further research and strategy development to treat human diseases.

Similar to ATRA that signals through intracellular receptors, lipids extracted from *T. cruzi* parasites induce alternative activation of macrophages and counteract inflammatory responses (67). The activation of PPAR (peroxisome proliferator activator receptor) γ signaling pathway by parasite lipids might reduce NF-κB pathway and prevent M1 responses (67). Likewise, the PPARα ligand fenofibrate induces a pro-repair M2 response during acute and chronic *T. cruzi* infection (68, 69). Furthermore, treatment with fenofibrate reduces inflammation, fibrosis and biomarkers of tissue damage, and improves heart functioning in experimental Chagas disease in a macrophage dependent fashion (68, 69). Interestingly, a short-term treatment of chronically infected mice with the betulinic acid derivative BA5 helped to prevent inflammation and fibrosis by inducing IL-10 and M2 polarization (70). Treatment with BA5 did not change parasite burden but could be associated to current anti-parasite drugs as an anti-inflammatory therapy (70). How to apply these new anti-inflammatory tools to prevent pathology in Chagas disease is a path yet to be explored.

## 5 Pharmacologically targeting M1 and M2 macrophages

M1 macrophages play a protective role during acute *T. cruzi* infection by phagocytosing parasites released from disrupted infected cells, followed by parasite killing within macrophages (11, 12). By contrast, M2-like macrophages harbor and fuel parasite infection, by diverting L-arginine metabolism towards the polyamine pathway (37, 71). Moreover, delayed induction of protective M1 responses can contribute to parasite dissemination and disease (12), whereas exacerbated inflammation ensues pathology. Therefore, the mechanisms that govern M1 and M2 macrophage phenotypes are potential targets for immunomodulation to improve immunity or downregulate pathogenic inflammatory responses (**Figure 1B**).

In *T. cruzi* infected B6 mice, PLA_2 (_phospholipase A_2_) and PI3K (phosphatidyl inositol 3 kinase) signaling pathways induce macrophage activation and protective immunity, while genetic ablation and pharmacological inhibition promote a shift to M2 macrophages and result in increased parasitemia and parasite load in the heart, associated with heart pathology/defective function (72, 73). By contrast, regulatory mechanisms such as SLAMF1 (signaling lymphocytic activation molecule) that reduces NADPH (nicotinamide adenine dinucleotide phosphate) oxidase and CD73 ectonucleotidase downregulate macrophage activation in susceptible BALB/c mice and are potential targets to improve macrophage-mediated immunity towards M1 responses (74, 75). Importantly, CD73 ablation and pharmacological inhibition prevented heart pathology and arrhythmia associated with parasite infection, tissue damage and inflammation (75, 76).

Association between M2 macrophages and susceptibility to *Leishmania* parasites (37–40, 42, 53) indicate that macrophage phenotypes might be targets for immunotherapy in Leishmaniasis. The L-arginine metabolism through the Arg1 activity is a hallmark of diffuse cutaneous Leishmaniasis in patients (37, 77, 78). In experimental models, susceptibility versus resistance to *L. major* infection correlates well with increased Arg1 expression and Th2 responses in BALB/c versus B6 mice (79). Inhibition of Arg1 activity helped both parasite and lesion control in *L. major*-infected BALB/c mice (79). Conversely, treatment with L-ornithine increased susceptibility in otherwise resistant B6 mice (79). In *T. cruzi* infection, IL-13-induced susceptibility is associated with enhanced M2 responses, such as Arg1 activity, whereas treatment with Arg1 inhibitors reduced mortality (80). Accordingly, infection of BMDMs with virulent but not less virulent *T. cruzi* parasites subverts parasite killing by inducing Arg1 expression and downmodulating iNOS expression (81).

In addition to L-arginine metabolism, other aspects of immunometabolism are potential targets for the control of macrophage plasticity and *Leishmania* infection (82, 83). Iron containing nanoparticles target host cell metabolism and improve protective M1 responses to fight *Leishmania* parasites (8). Induction versus inhibition of glucose-6-phosphate dehydrogenase (G6PDH) activity regulates NO-dependent resistance versus macrophage susceptibility to *Leishmania* parasites (84).

*T. cruzi* infection induces the metabolic check point mammalian Target of Rapamycin inhibition (mTOR) mTORC1 pathway in macrophages (85). Moreover, *in vitro* treatment with the mTOR inhibitor rapamycin reduced M2 responses, increased proinflammatory cytokines, and promoted parasite control in a NLRP3-dependent fashion (85). How to regulate immunometabolism *in vivo* in a cell specific fashion is a challenge to develop successful therapy that prevents homeostasis disruption.

*T. cruzi* infection modifies macrophage miRNA responses (86) and some miRNAs control macrophage plasticity to induce M1 and M2 phenotypes (87). In macrophages infected with antimony-resistant *Leishmania* parasites, certain miRNAs downmodulate iNOS expression and subvert Myd88 (myeloid differentiation primary response 88)-NFκB signaling to promote early IL-10 secretion that contributes to increased parasite burden and pathology in visceral Leishmaniasis (88). Remarkably, modulation of miRNAs can be used *in vivo* and are potential tools to shape macrophage phenotypes and ability to control *Leishmania* infection (89, 90).

## 6 Identifying new inhibitable pro-M2 molecular targets

During infection, M2 macrophages are parasite-permissive host cells that also play a role in anti-inflammatory responses, tissue remodeling, and fibrosis (17, 49). Macrophages respond to Th2 cytokines and to recognition and removal of apoptotic cells (efferocytosis) by turning off M1 and switching to pro-M2 signaling pathways (17, 71). A major goal on drug discovery and development of host-directed therapies is to identify new selective targets that show anti-parasite potential without enhancing pathology or disrupting host homeostasis. i.e. tissue repair (68, 69) (**Figure 1B**).

We previously showed that T cell apoptosis increases during *T. cruzi* infection and contributes to defective T cell responses that might underly parasite persistence (91). Molecular mechanisms such as ligands, death receptors, and the components of proapoptotic machinery were studied and tested in proof-of-concept experiments in acute *T. cruzi* infection (92). By summarizing, treatment with anti-FasL and the pan caspase inhibitor zVAD improved both T-cell and macrophage-mediated immunity and reduced parasitemia during acute infection (93–95). Nonetheless, we observed a timely regulated increase in Th1 and Th2 responses in FasL deficient or anti-FasL treated mice (93, 96), and that caspase-8 deficiency also upregulated Th2 responses to *T. cruzi* and *L. major* infections (97, 98). Therefore, whereas interesting as a hypothesis test, interrupting apoptosis-inducing signaling might disrupt homeostasis and bring considerable concern issues. Nonetheless, a vaccine strategy prevented the induction of Fas-expressing proapoptotic CD8 T cells after *T. cruzi* challenge (99), opening a safer prophylaxis avenue than pharmacological targeting of apoptosis signaling pathways. Importantly, vaccine-induced CD8 T cells exhibit effector responses and differ from exhausted/proapoptotic T cells generated during *T. cruzi* infection (99), which might fail to induce early macrophage activation to control infection (12).

Upon apoptosis, efferocytosis removes apoptotic cells and prevents the release of DAMPs and subsequent inflammation. Multiple receptors detect phosphatidylserine exposure or other apoptosis features and initiate phagocytosis of apoptotic cells and anti-inflammatory signaling to ensure homeostasis (100–103). During inflammation, however, macrophages might use a different set of efferocytosis receptors providing an opportunity for selective pharmacological intervention. Accordingly, anti-inflammatory versus inflammatory stimuli induce preferential expression of the TAM (Tyro Axl Mer) receptors Mer versus Axl in macrophages (104).

Efferocytosis of apoptotic cells promotes *T. cruzi* replication within macrophages in a TGF-β, prostaglandin E_2_, and polyamine dependent fashion (71). In peritoneal macrophages from infected mice, the integrin αvβ3 was identified as a putative efferocytosis receptor for apoptotic cell-inducing signaling that contributes to *T. cruzi* growth (71). For addressing the role of efferocytosis receptors during parasite infection, we used single Mer or Axl defective mice and BMDMs cultured with T cells from *T. cruzi*-infected mice, which provided both effector and pro-apoptotic cells able to impact on macrophage phenotypes (105). *In vitro*, Mer deficiency significantly reduced efferocytosis but had little impact on macrophage phenotype (105). Remarkably, Axl defective macrophages showed improved M1 responses, such as CXCL9 and IL-12p35 expression, iNOS expression and NO production, and increased ability to control *T. cruzi* infection despite only partial inhibition of efferocytosis (105).

Moreover, Axl-deficient mice had reduced peak parasitemia and less inflammation and fibrosis in their hearts compared to infected B6 WT and Mer^-/-^ mice (105). Infected Axl^-/-^ mice also showed increased M1 responses in the peritoneum and spleen and iNOS expression in the heart (105). These results indicate that Axl is a selective target to improve macrophage-mediated immunity without interfering with apoptosis or Mer-mediated homeostatic efferocytosis. Nonetheless, the accumulation of apoptotic cells in infected Axl-deficient mice (105) is a potential deleterious side effect that deserves caution in efferocytosis inhibition.

During *Leishmania* infection, the TAM receptor Mer plays a role in the efferocytosis of infected neutrophils by DCs and suppression of T cell responses (106). Furthermore, Mer-mediated efferocytosis of infected neutrophils transfers *Leishmania* parasites to macrophages (107). Dual Mer/Axl genetic ablation reduced the development of M2 macrophages and parasite infection (107). Nonetheless, increased lesions in infected double KO mice indicate that Mer and/or Axl play an essential anti-inflammatory role to prevent parasite-induced pathology (107). New studies in single-receptor defective mice might clarify the individual roles of TAM receptors in *L. major* infection.

## 7 Concluding remarks

Targeting immunoregulatory host mechanisms such as T-cell coinhibitory receptors (51, 108) can improve otherwise suppressed immune responses or upregulate immunity. Likewise, unveiling the mechanisms of macrophage plasticity (87) might translate into host-directed therapies to mitigate human diseases. New drug delivery systems by using liposomes or nanoparticles (8) and vaccine mRNA technology will foster the development of new drugs, vaccines, and therapeutic vaccines to fight infectious diseases. How these remarkable scientific and technological advances (**Table 1**) might translate into clinical trials for Chagas disease and Leishmaniasis and lead to effective solutions for tropical neglected diseases will demand major scientific, industrial, and political efforts.

**Table 1:** Macrophage plasticity: molecular targets to shape M1 and M2 phenotypes.

Molecular target	Intervention/experimental model	Macrophage findings	Infection and pathology outcome	Ref. n°
RANKL-RANK	RANKL + low IFN-γ/inflammatory pMacs RANK-Fc- treated CD40L KO mice	Switch M2-M1 Reduced IL-12 producing cells	NO/ROS-dependent *L. major* killing Increased *L. major* infection, increased lesion	(60, 62)
APRIL/BAFF-TACI	APRIL or BAFF-treated pMacs TACI KO mice WT Mac transfer into TACI KO mice	Reduced M2 responses M2 responsesReduced M2 responses	Reduced *in vitro L. major* infection Increased *L. major* infection/lesion Reduced *L. major* infection, reduced lesion	(65)
Th2 cytokine/Arg1-polyamine pathway	IL-13 tg mice Arg1 inhibitor/IL-13 tg Arg1 inhibitor/BALB/c Ornithine/B6 mice	Increased M2 response/Arg1	Increased *T. cruzi* infection, increased mortality Reduced mortality Reduced *L. major* infection/lesion Increased *L. major* infection/lesion	(79, 80)
ATRA-RXR/RAR	Paw injection/B6 mice Ip injection in B6/BALB/c mice	Reduced NO responses Reduced M1 responses	Increased *L. major* infection, increased lesion Increased *L. major* load in pMacs	(29, 66)
Fenofibrate-PPAR-α	Oral gavage/acute (B6) and chronic (BALB/c) *T. cruzi* infection	Increased M2 and reduced M1 responses	Reduced inflammation, heart fibrosis and tissue damage, improved heart function.	(68, 69)
CD73 ecto-nucleotidase	CD73 KO/acute *T. cruzi* infection Iv CD73 inhibitor/BALB/c mice	CD73^-/-^ M1-like heart Macs Switch M2-M1	Reduced parasite burden, increased parasitemia Reduced tissue parasitism, tissue damage, improved heart function	(75, 76)
SLAMF1	SLAMF1 KO mice/ acute *T. cruzi* infection Anti-SLAMF1/BALB	Reduced M2-like heart Macs	Reduced tissue parasitism, mortality, and tissue damage Reduced tissue parasitism	(74)
TAM receptors	Axl KO mice/ acute *T. cruzi* infection Mer/Axl DKO mice/*L. major* infection	Axl^-/-^ M1-like heart iNOS^+^ cell Switch M2-M1	Reduced parasitemia, inflammation and fibrosis Reduced *L*. major infection, increased lesions	(105, 107)
PI3Kγ- AKT1 signaling	PI3K inhibitor/BMDM Sc PI3K inhibitor/B6 mice PI3Kγ KO mice/ acute *T. cruzi* infection AKT1-Lys KO mice	PI3Kγ^-/-^ M2-like Macs	Increased *T. cruzi* infection Increased weight loss and mortality Increased tissue parasitism, tissue damage, inflammation, mortality High tissue parasitism, mortality	(73)
PLA_2_β	PLA_2_β KO mice/acute *T. cruzi* infection	PLA_2_β ^-/-^ M2-like Macs	High tissue parasitism	(72)
miR146a-5p	Anti-146a oligos/BMDMs Iv anti 146a oligos/BALB/c mice	Switch M2-M1 responses Switch M2-M1 responses	Reduced *L. donovani* phagocytosis, reduced parasite survival Reduced parasite burden in *L. donovani* infection	(89)
